# Supplementary material for: One-Step Rapid and Sensitive ASFV p30 Antibody Detection via Nanoplasmonic Biosensors
Source: Microbiol Spectr. 2022 Oct 31;10(6):e02343-22. doi: 10.1128/spectrum.02343-22 (PMC9769802; doi:10.1128/spectrum.02343-22)
Supplement: Supplemental file 1 — Table S1, Fig. S1-S12. Download spectrum.02343-22-s0001.pdf, PDF file, 2.5 MB [file spectrum.02343-22-s0001.pdf]

Table S1.Background (strong/weak or negative) of 186 clinical sera

Clinical serum Sample Number	The ASFV ELISA P72 enzyme-linked immunosorbent antibody detection kit（blocking rate%,X%⩾50%,ASFV positive;X%⩽40%,ASFV negative;50%⩾X%⩾40%,Suspected samples)	The ASFV P30 antibody detection Nanoplasmonic sensors（relative OD change)
1#	98.21	0.378
2#	96.33	0.3708
3#	96.8	0.3563
4#	96.75	0.3559
5#	96.58	0.3379
6#	96.25	0.3375
7#	95.68	0.3349
8#	95.67	0.3336
9#	96.25	0.3142
10#	96.88	0.2766
11#	97.58	0.2739
12#	95.63	0.2716
13#	95.43	0.2664
14#	94.85	0.2626
15#	95.78	0.2589
16#	95.12	0.2573
17#	94.55	0.2488
18#	94.87	0.2449
19#	94.68	0.2409
20#	94.5	0.2386
21#	96.58	0.2347
22#	94.86	0.2205
23#	94.85	0.2142
24#	95.28	0.2112
25#	95.68	0.2069
26#	93.88	0.2047
27#	93.24	0.203
28#	93.67	0.2025
29#	93.55	0.201
30#	93.65	0.196
31#	93.54	0.1958
32#	93.51	0.1941
33#	93.4	0.1905
34#	93.17	0.1877
35#	93.02	0.1817
36#	93.27	0.1801
37#	92.15	0.1794
38#	91.89	0.1788
39#	92.1	0.1773
40#	92.55	0.1748
41#	93.15	0.1745
42#	92.11	0.1709
43#	92.08	0.1701
44#	92.01	0.1641
45#	91.88	0.1634

46#	91.58	0.1555
47#	91.86	0.155
48#	86.25	0.1528
49#	85.24	0.1505
50#	86.12	0.1504
51#	85.02	0.1497
52#	85.24	0.145
53#	85.34	0.1378
54#	85.01	0.135
55#	84.55	0.134
56#	84.37	0.1301
57#	83.05	0.1295
58#	79.81	0.1289
59#	79.02	0.1279
60#	79.04	0.1263
61#	79.55	0.1259
62#	78.24	0.1257
63#	78.99	0.1242
64#	78.56	0.1233
65#	78.45	0.123
66#	78.88	0.1217
67#	78.45	0.1185
68#	78.24	0.1128
69#	79.1	0.1123
70#	78.02	0.1119
71#	78.24	0.1113
72#	78.25	0.1091
73#	78.14	0.107
74#	78.95	0.1036
75#	78.65	0.1035
76#	78.54	0.1025
77#	77.56	0.0963
78#	77.25	0.094
79#	77.12	0.0931
80#	77.35	0.0919
81#	77.25	0.0912
82#	76.25	0.0911
83#	76.35	0.0907
84#	75.28	0.089
85#	75.89	0.0881
86#	75.68	0.0876
87#	74.25	0.0864
88#	74.18	0.0857
89#	74.58	0.0836
90#	74.68	0.0788
91#	74.12	0.0784
92#	73.25	0.0778
93#	72.48	0.0761
94#	70.15	0.0753
95#	70.12	0.0747
96#	70.25	0.0745
97#	70.48	0.0741
98#	68.25	0.074

99#	67.52	0.0727
100#	65.45	0.0708
101#	66.25	0.0705
102#	64.24	0.069
103#	63.28	0.0684
104#	62.88	0.0646
105#	61.49	0.0644
106#	61.25	0.0592
107#	60.25	0.058
108#	61.11	0.0579
109#	61.25	0.0563
110#	61.3	0.0552
111#	61.02	0.0537
112#	60.25	0.0536
113#	60.38	0.0515
114#	60.58	0.0492
115#	60.45	0.0486
116#	59.88	0.048
117#	59.7	0.0461
118#	59.45	0.0437
119#	59.4	0.0395
120#	59.12	0.0394
121#	58.99	0.0386
122#	58.39	0.0386
123#	58.37	0.0374
124#	58.36	0.0372
125#	57.23	0.0365
126#	56.55	0.0361
127#	56.14	0.0354
128#	56.34	0.0353
129#	56.15	0.0347
130#	54.28	0.0345
131#	55.16	0.0315
132#	54.66	0.0299
133#	53.2	0.0238
134#	53.11	0.0216
135#	53.1	0.0203
136#	52.14	0.016
137#	51.2	0.0143
138#	51.85	0.013
139#	51.25	0.0107
140#	50.45	-0.0278
141#	50.14	-0.0438
142#	50.18	-0.0499
143#	50.68	-0.073
144#	50.12	-0.0611
145#	50.44	-0.0568
146#	50.15	-0.0748
147#	3.12	-0.0539
148#	3.12	-0.0324
149#	3.18	-0.0384
150#	3.28	-0.0329
151#	3.46	-0.0415

152#	3.78	-0.0279
153#	3.98	-0.0319
154#	4.15	-0.0308
155#	4.65	-0.0308
156#	4.28	-0.0298
157#	4.12	-0.0273
158#	6.58	-0.0351
159#	6.78	-0.0199
160#	6.45	-0.0229
161#	7.58	-0.0356
162#	7.56	-0.029
163#	7.98	-0.0389
164#	8.25	-0.0265
165#	2.88	-0.0308
166#	3.56	-0.0306
167#	8.47	-0.0298
168#	7.12	-0.0264
169#	7.25	-0.0287
170#	3.68	-0.0311
171#	7.12	-0.0324
172#	2.18	-0.0412
173#	4.38	-0.0298
174#	2.02	-0.0378
175#	3.34	-0.0311
176#	3.46	-0.0318
177#	7.68	-0.0308
178#	6.18	-0.0321
179#	5.06	-0.0298
180#	2.01	-0.0412
181#	3.68	-0.0287
182#	4.98	-0.0302
183#	3.88	-0.0287
184#	4.67	-0.0261
185#	4.89	-0.0265
186#	4.98	-0.0271

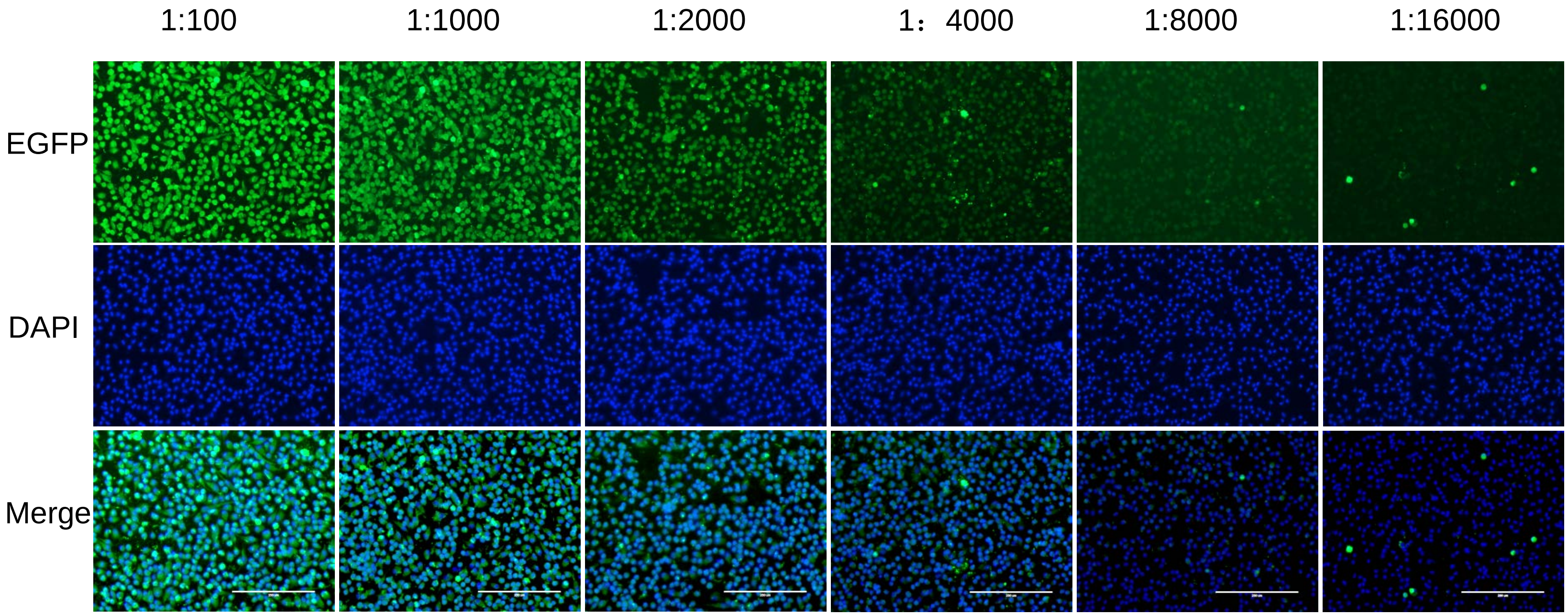

Figure S1.Primary PAMs were seeded in 48-well plates and infected with ASFV at 0.1 multiplicity of infection (MOI) for 48 hpi, 1:100–1:16000 dilution of the ASFV-positive serum as the primary antibody, and fluorescence microscopy was performed using an anti-pig antibody (green). DAPI was used to stain the nucleus (blue). Images are representative of three independent experiments. Scale bar, 200 μM.

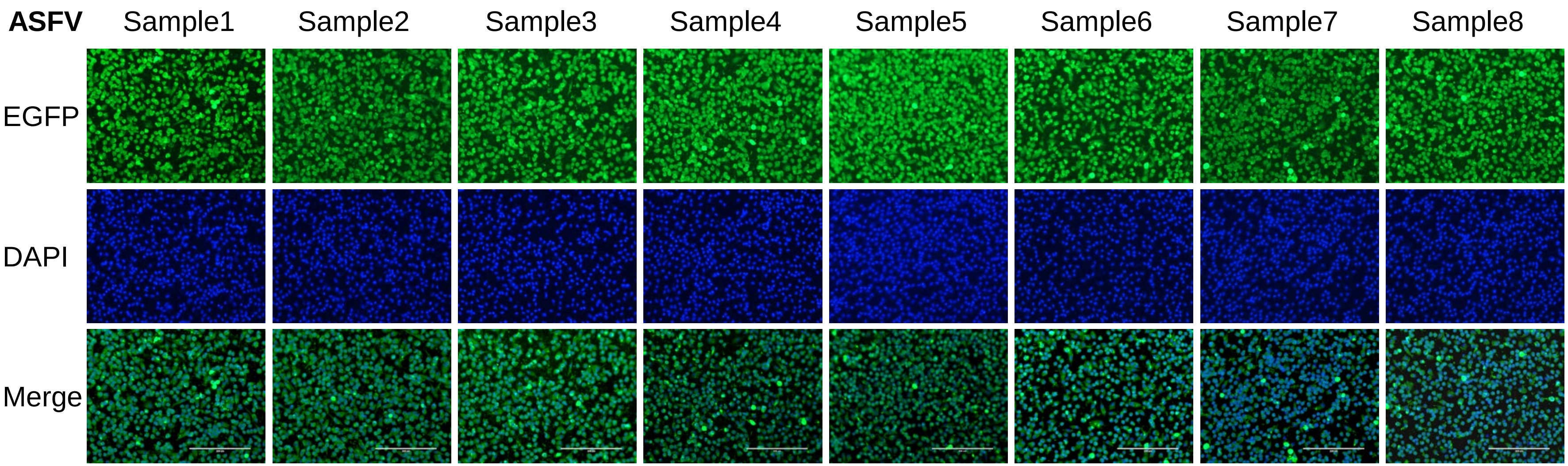

Figure S2. Primary PAMs were seeded in 48-well plates and infected with ASFV at 0.1 multiplicity of infection (MOI) for 48hpi, 1:100 dilution of the ASFV positive serum as primary antibody, and fluorescence microscopy was performed using an anti-pig antibody (green) .DAPI was used to stain for the nucleus (blue). Scale bar, 200 μM.

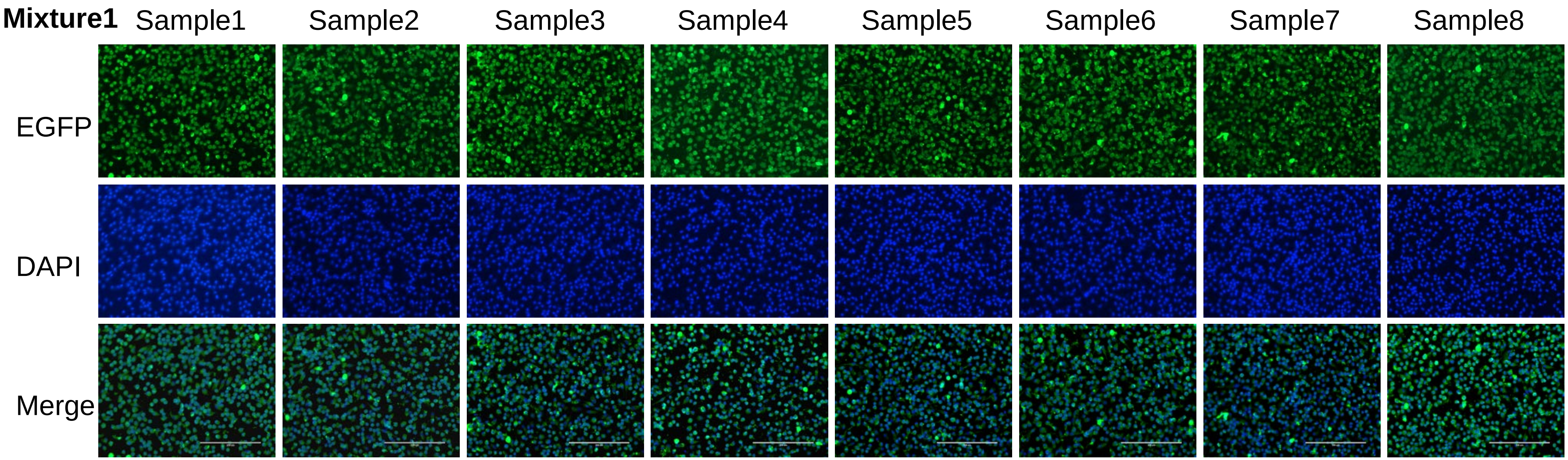

Figure S3. Primary PAMs were seeded in 48-well plates and infected with ASFV at 0.1 multiplicity of infection (MOI) for 48hpi, 1:100 dilution of the mixture 1 group (including ASFV, PRRSV, CSFV, PCV-2, PEDV, TGEV, JEV, H1N1, PRV) serum as primary antibody, and fluorescence microscopy was performed using an anti-pig antibody (green) .DAPI was used to stain for the nucleus (blue). Scale bar, 200 μM.

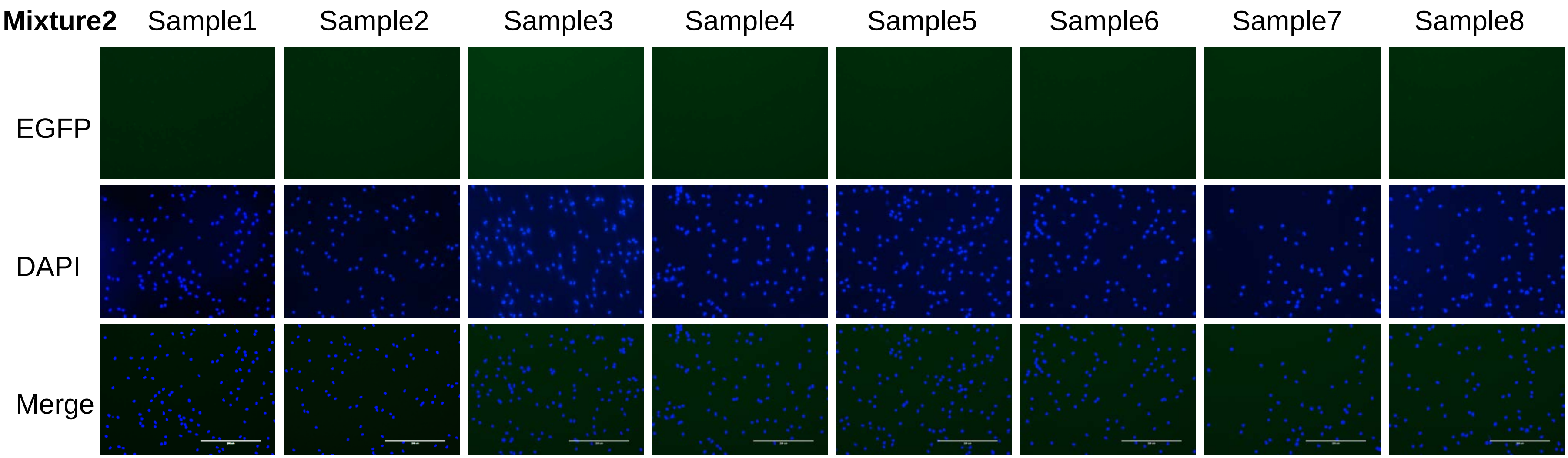

Figure S4. Primary PAMs were seeded in 48-well plates and infected with ASFV at 0.1 multiplicity of infection (MOI) for 48hpi, 1:100 dilution of the mixture 2 group (including PRRSV, CSFV, PCV-2, PEDV, TGEV, JEV, H1N1, PRV) serum as primary antibody, and fluorescence microscopy was performed using an anti-pig antibody (green) .DAPI was used to stain for the nucleus (blue). Scale bar, 200 μM.

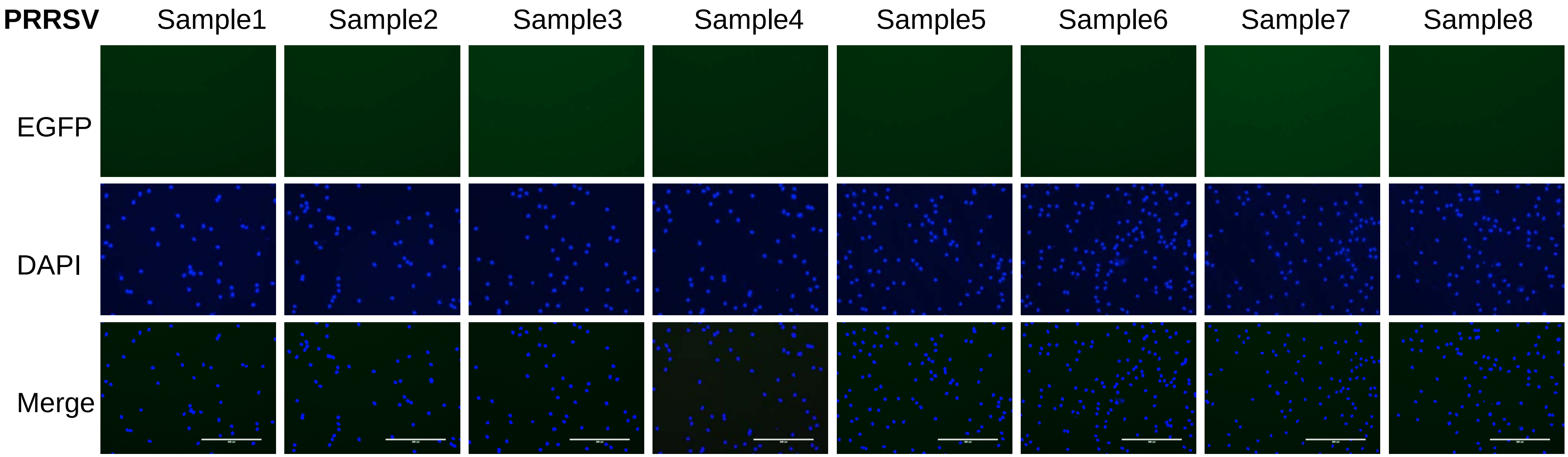

Figure S5. Primary PAMs were seeded in 48-well plates and infected with ASFV at 0.1 multiplicity of infection (MOI) for 48hpi, 1:100 dilution of the PRRSV positive serum as primary antibody, and fluorescence microscopy was performed using an anti-pig antibody (green) .DAPI was used to stain for the nucleus (blue). Scale bar, 200 μM.

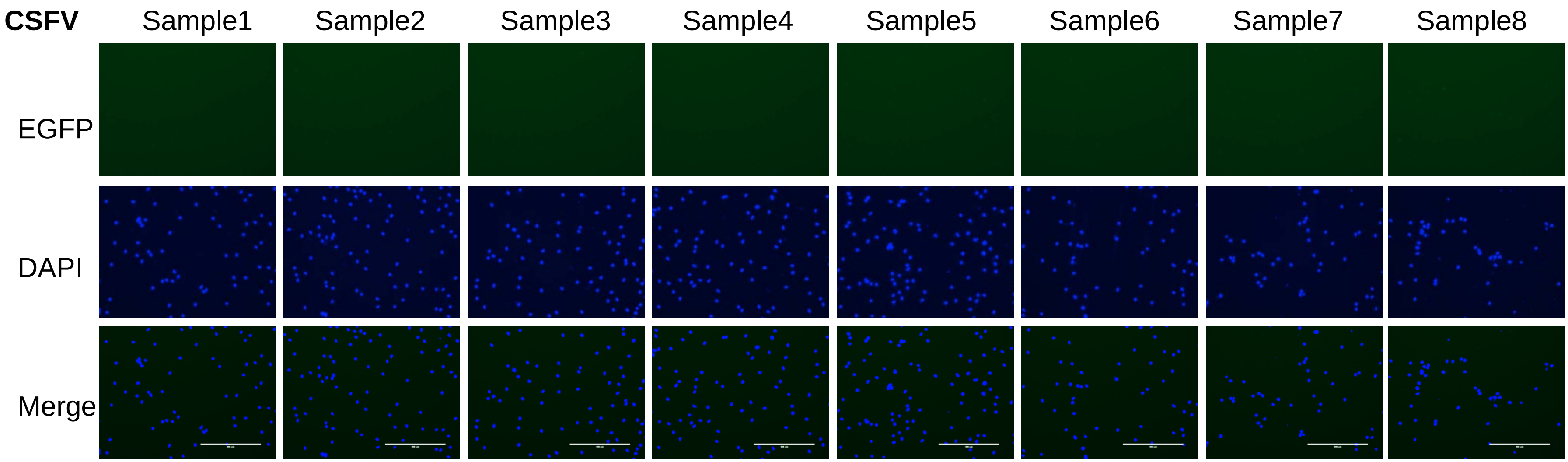

Figure S6. Primary PAMs were seeded in 48-well plates and infected with ASFV at 0.1 multiplicity of infection (MOI) for 48hpi, 1:100dilution of the CSFV positive serum as primary antibody, and fluorescence microscopy was performed using an anti-pig antibody (green) .DAPI was used to stain for the nucleus (blue). Scale bar, 200 μM.

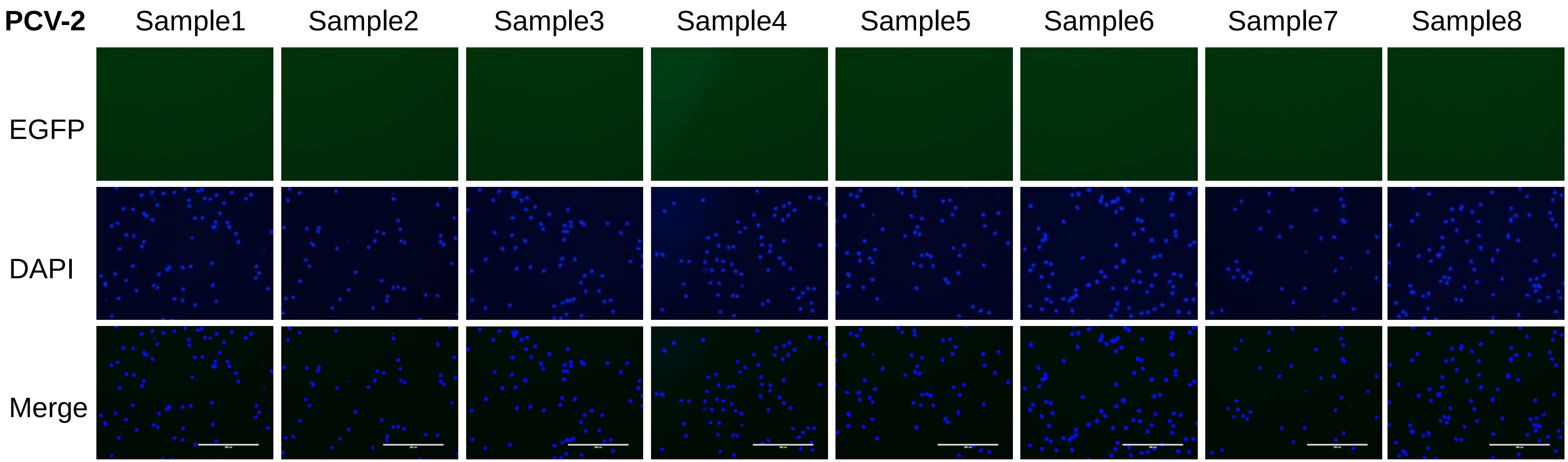

Figure S7. Primary PAMs were seeded in 48-well plates and infected with ASFV at 0.1 multiplicity of infection (MOI) for 48hpi, 1:100 dilution of the PCV-2 positive serum as primary antibody, and fluorescence microscopy was performed using an anti-pig antibody (green) .DAPI was used to stain for the nucleus (blue). Scale bar, 200 μM.

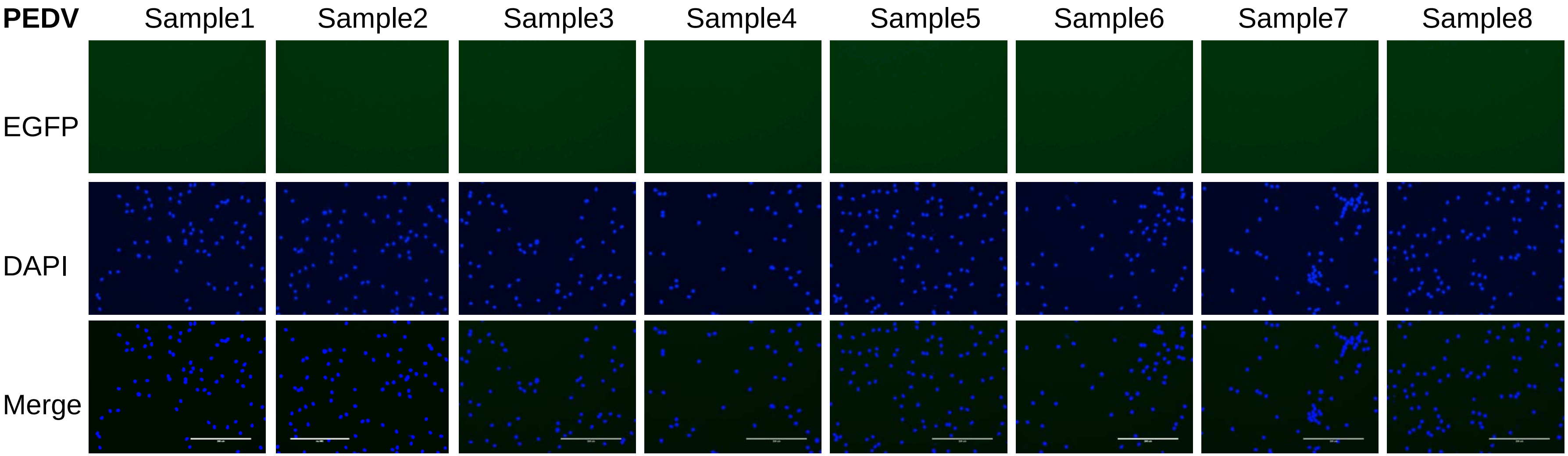

Figure S8. Primary PAMs were seeded in 48-well plates and infected with ASFV at 0.1 multiplicity of infection (MOI) for 48hpi, 1:100 dilution of the PEDV positive serum as primary antibody, and fluorescence microscopy was performed using an anti-pig antibody (green) .DAPI was used to stain for the nucleus (blue). Scale bar, 200 μM.

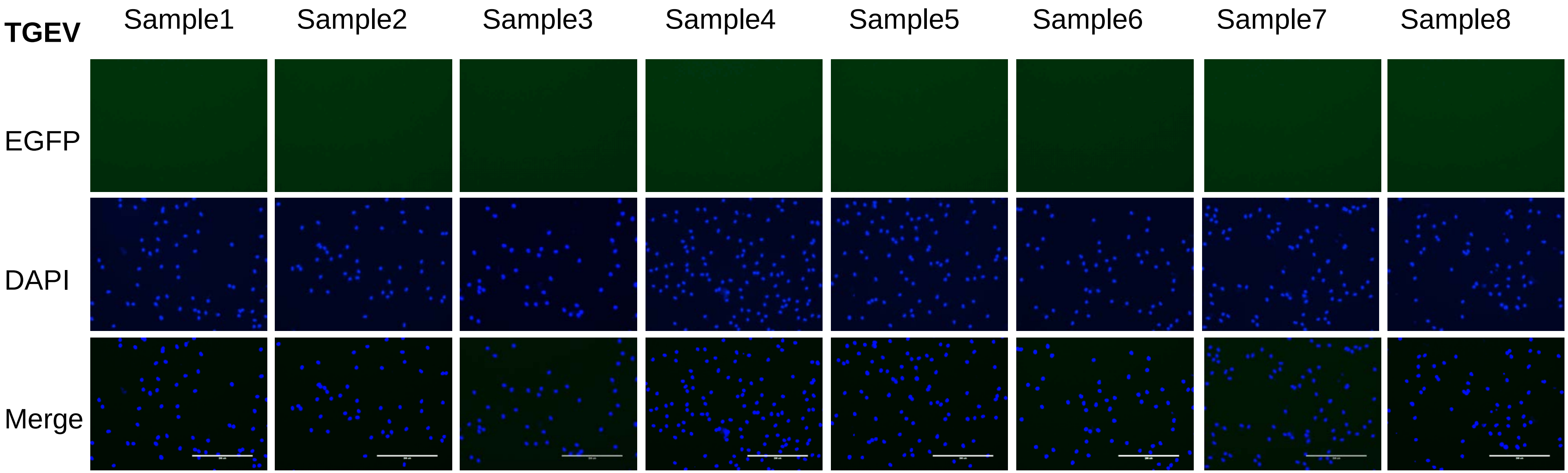

Figure S9. Primary PAMs were seeded in 48-well plates and infected with ASFV at 0.1 multiplicity of infection (MOI) for 48hpi, 1:100 dilution of the TGEV positive serum as primary antibody, and fluorescence microscopy was performed using an anti-pig antibody (green) .DAPI was used to stain for the nucleus (blue). Scale bar, 200 μM.

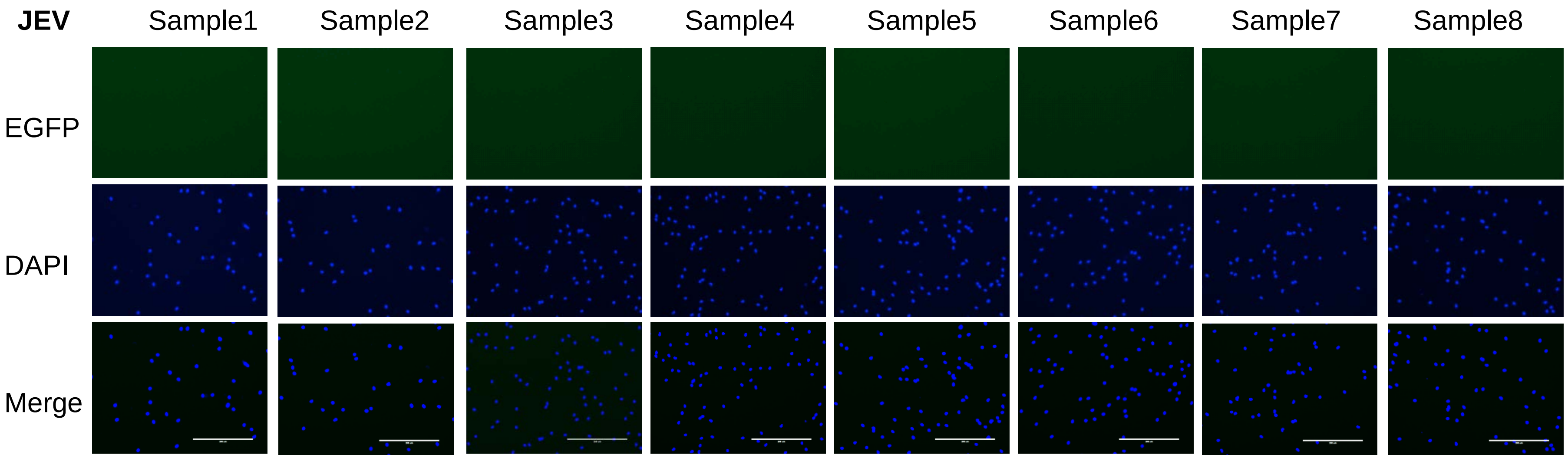

Figure S10. Primary PAMs were seeded in 48-well plates and infected with ASFV at 0.1 multiplicity of infection (MOI) for 48hpi, 1:100 dilution of the JEV positive serum as primary antibody, and fluorescence microscopy was performed using an anti-pig antibody (green) .DAPI was used to stain for the nucleus (blue). Scale bar, 200 μM.

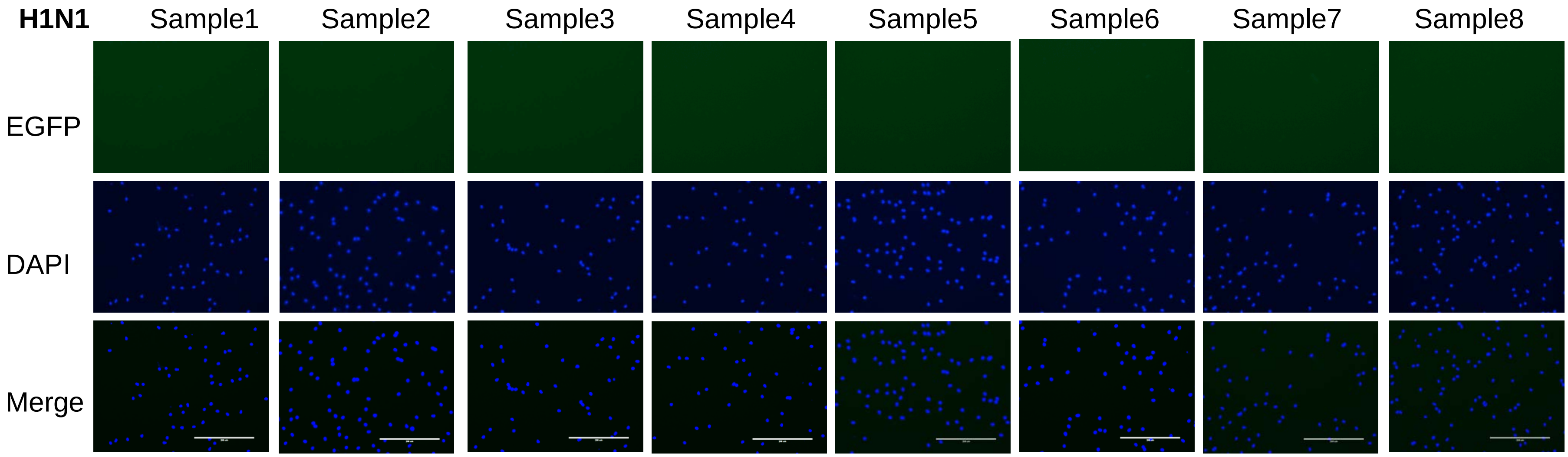

Figure S11. Primary PAMs were seeded in 48-well plates and infected with ASFV at 0.1 multiplicity of infection (MOI) for 48hpi, 1:100 dilution of the H1N1 positive serum as primary antibody, and fluorescence microscopy was performed using an anti-pig antibody (green) .DAPI was used to stain for the nucleus (blue). Scale bar, 200 μM.

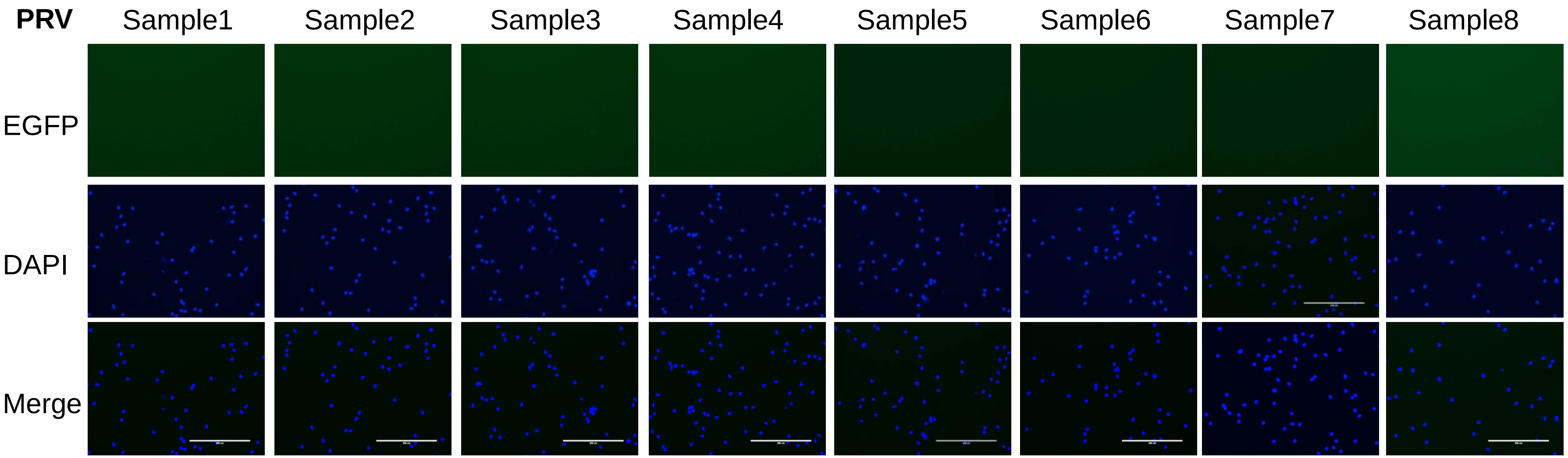

Figure S12. Primary PAMs were seeded in 48-well plates and infected with ASFV at 0.1 multiplicity of infection (MOI) for 48hpi, 1:100 dilution of the PRV positive serum as primary antibody, and fluorescence microscopy was performed using an anti-pig antibody (green) .DAPI was used to stain for the nucleus (blue). Scale bar, 200 μM.
